# Correction: Arriero-Cabañero et al. Transplantation of Predegenerated Peripheral Nerves after Complete Spinal Cord Transection in Rats: Effect of Neural Precursor Cells and Pharmacological Treatment with the Sulfoglycolipid Tol-51. *Cells* 2024, *13*, 1324

**DOI:** 10.3390/cells15030249

**Published:** 2026-01-28

**Authors:** Alejandro Arriero-Cabañero, Elisa García-Vences, Stephanie Sánchez-Torres, Sergio Aristizabal-Hernandez, Concepción García-Rama, Enrique Pérez-Rizo, Alfonso Fernández-Mayoralas, Israel Grijalva, Vinnitsa Buzoianu-Anguiano, Ernesto Doncel-Pérez, Jörg Mey

**Affiliations:** 1 Laboratorio de Regeneración Neural, Hospital Nacional de Parapléjicos, 45071 Toledo, Spain; aarrieroc@externas.sescam.jccm.es (A.A.-C.); searhe@hotmail.com (S.A.-H.); concepciongarciarama@gmail.com (C.G.-R.); jmey@sescam.jccm.es (J.M.); 2 Facultad de Ciencias de la Salud, Centro de Investigación en Ciencias de la Salud (CICSA), Universidad Anáhuac México Norte, Huixquilucan 52786, Mexico; edna.garcia@anahuac.mx; 3 Secretaría de la Defensa Nacional, Escuela Militar de Graduados en Sanidad, Ciudad de Méxcio 11200, Mexico; 4 Instituto Mexicano del Seguro Social, Unidad de Investigación Médica en Enfermedades Neurológicas, Hospital de Especialidades, Centro Médico Nacional Siglo XXI. Av. Cuauhtémoc 330, Col. Doctores, Mexico City 06720, Mexico; stephanie.sanchez.torres@gmail.com (S.S.-T.); igrijalvao@yahoo.com (I.G.); 5 Unidad de Ingeniería y Evaluación Motora del Hospital Nacional de Parapléjicos, 45071 Toledo, Spain; enriquep@sescam.jccm.es; 6 Instituto de Química Orgánica General, CSIC, Juan de la Cierva 3, 28006 Madrid, Spain; alfonso.mayoralas@csic.es; 7 EURON Graduate School of Neuroscience, 6229ER Maastricht, The Netherlands

In the original publication [1], there were errors in Table 1, Figure 5 and Section 3.1. 

In Table 1, there was an error regarding the number of animals that were lost during the different phases of the experiments. The corrected Table 1 is below.

In Figure 5, the position of the PPN implant was not indicated correctly below the *x*-axis. The corrected Figure 5 is below:

In Section 3.1, there was an error in the last phrase of the first paragraph regarding the number of animals evaluated at the end of the study. The correct text is below:

To assess axonal regeneration reliably, we made complete spinal cord transections at thoracic level T9. This is a very severe lesion, but it is considered necessary to test spinal reconnection with combinatorial therapy of biomaterials, cell transplantation, and pharmacological agents [30,36,37]. Despite intensive postoperative care, this disease model is characterized by high mortality, specifically during the first month after SCI (Table 1). No animals were lost due to the surgery per se, and after transplantation, there was no indication of sickness behavior or rejection of the transplanted tissue. In the following weeks, eight rats had to be sacrificed due to humane endpoints (urinary infection and self-mutilation). At the designated end of the study, 27 animals were evaluated (Table 1, Figure 1).

The authors state that the scientific conclusions are unaffected. This correction was approved by the Academic Editor. The original publication has also been updated.

## Figures and Tables

**Figure 5:**
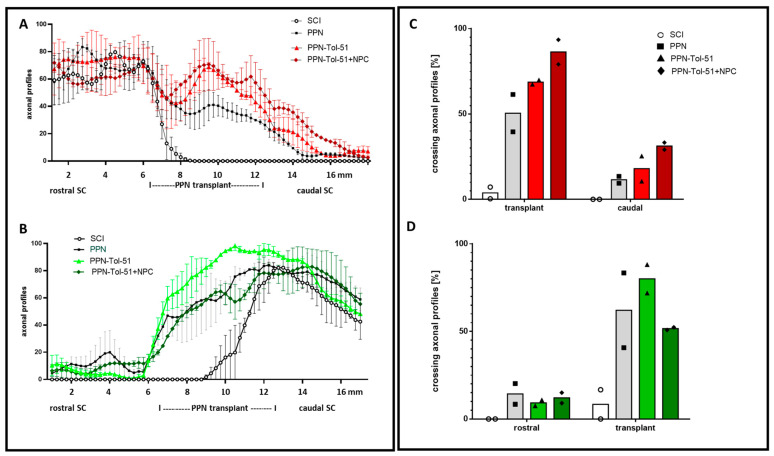
Quantification of axonal regeneration in the SCI group and PPN graft groups. In the left panel, the graphs show the percentage of tracer-labeled axons in the sections counted at 250 μm intervals; mean +/− SD and *n* = 2 rats/group (**A**,**B**). Axons were labeled with tracer injection from the motor cortex ((**A**), red) and from the lumbar spinal cord ((**B**), green). Regenerative axonal profiles were found within the PPN implants but not in the corresponding area of the SCI group. In panel (**C**), the number of regenerating axons within the transplant site and in the spinal cord caudal to the injury site was normalized to the number of labeled axons rostral to the injury site (i.e., in the rostral segment of the spinal cord for tracing axons from the cortex area). In bar chart (**D**): Quantification of axonal regeneration with axonal tracing of the lumbar spinal cord (using trace data from the caudal section of the spinal cord for normalization). A minority of regenerating axons found within the PPN implants continued to grow into the distal segments of the spinal cord. This percentage was greater for treatment with (PPN+Tol-51+NPC) for the anterior tracer (**C**) and for treatment with (PPN+Tol-51) for the posterior tracer (**D**). The columns show the average of two rats/group.

**Table 1:** Loss of experimental animals due to postsurgical complications and humane endpoints.

Experimental Groups	SCI	SCI+Alk-Fibrin	PPN	PPN+Tol-51	PPN+Tol-51+NPC
*n* (total = 62)	12	12	12	12	14
Animals lost after SCI:					
Before transplant	6	6	5	7	3
Animals lost after transplant surgery:					
Month 1	1	0	2	1	2
Month 2	0	0	0	0	2
Month 3	0	0	0	0	0
Month 4	0	0	0	0	0
Final group size:					
	SCI	SCI+Alk+Fibrin	PPN	PPN+Tol-51	PPN+Tol-51+NPC
*n* (total = 27)	5	6	5	4	7
